# Standardized sarcopenia assessment should be incorporated into prognosis analysis of esophageal cancers: a prospective cohort study lasting 8 years

**DOI:** 10.3389/fnut.2026.1865456

**Published:** 2026-06-12

**Authors:** Qi Liu, Yongkui Yu, Xiankai Chen, Xianben Liu, Wenqun Xing, Yin Li, Peiyu Wang

**Affiliations:** 1 Department of Thoracic Surgery, The Affiliated Cancer Hospital of Zhengzhou University & Henan Cancer Hospital, Zhengzhou, Henan, China; 2 Department of Thoracic Surgical Oncology, National Cancer Center/Cancer Hospital, Beijing, China; 3 Department of Thoracic Surgery, The First Affiliated Hospital of Zhengzhou University, Zhengzhou, Henan, China

**Keywords:** esophageal cancer, esophagectomy, sarcopenia, skeletal muscle, survival

## Abstract

**Background:**

The prognosis value of preoperative sarcopenia in esophageal cancer patients undergoing surgical resection have rarely been prospectively investigated. The necessity of a standardized sarcopenia assessment—including muscle mass, muscle strength, and physical performance—has not been tested.

**Methods:**

This prospective cohort study assessed the appendicular skeletal muscle mass index (ASMI), handgrip strength, and gait speed to classify participants as normal, pre-sarcopenia, or sarcopenia. The prognostic value of sarcopenia for overall survival (OS) and disease-free survival (DFS) was evaluated using propensity score matching, multivariable analysis, and subgroup analysis. The additive contribution of each muscle criterion to survival prediction was assessed using SHapley Additive exPlanations.

**Results:**

This study included 212 participants, with a median survivor follow-up of 91.8 months. Sarcopenia risk, including both pre-sarcopenia and sarcopenia, was identified as an independent prognostic factor for poor OS and DFS. These prognostic values remained significant across almost all patient subgroups. Incorporating the sarcopenia classification system into nomograms based on clinical cancer stage improved prediction efficacy for both OS and DFS. Additionally, although the three dimensions of muscle assessment were independently associated with OS and DFS, gait speed showed the largest contribution to survival prediction, while ASMI and handgrip strength showed comparably slightly inferior contributions.

**Conclusion:**

Sarcopenia assessment is recommended for incorporation into the prognosis analysis of surgically treated esophageal cancer patients. Comprehensive assessment of muscle mass, muscle strength, and physical performance is necessary for definitive diagnosis, severity classification, and effective prediction.

**Trial registration:**

Identifier, ChiCTR 1,800,017,792.

## Introduction

Esophageal cancer is the twelfth most common malignancy and the seventh leading cause of cancer-related death worldwide, representing a persistent threat to global public health (1). For locally advanced esophageal cancer, the standard treatment paradigm consists of neoadjuvant therapy followed by esophagectomy—a cornerstone of multidisciplinary management (2, 3). Despite advances in surgical techniques and systemic therapy, this patient population continues to experience poor long-term outcomes, with an overall 5-year survival rate less than 40% (4). This unsatisfactory clinical scenario underscores the urgent need to identify novel and modifiable prognostic factors to improve risk stratification and guide personalized management.

Sarcopenia, defined as the progressive loss of skeletal muscle mass accompanied by reduced muscle strength and physical performance (5, 6), has been widely recognized as a critical prognostic factor across various solid tumors (7, 8). Although accumulating evidence links sarcopenia to adverse outcomes in esophageal cancer (9–11), the current body of research is limited in two key aspects. First, there is a marked paucity of prospective cohort studies, with most investigations relying on retrospective analyses prone to selection bias and residual confounding, thereby limiting the ability to clarify the causal relationship between sarcopenia and clinical outcomes. Second, most retrospective studies diagnose sarcopenia solely based on muscle mass assessment, typically using the skeletal muscle area at the third lumbar vertebra level in computed tomography (CT) (9–11). These studies neglected the evaluation of muscle strength and physical performance—core components of sarcopenia as defined by international consensus guidelines (5, 6). This one-dimensional approach fails to capture the full clinical spectrum of sarcopenia, resulting in an incomplete understanding of its prognostic role in esophageal cancer.

Between 2018 and 2019, our research team established a prospective cohort of esophageal cancer patients undergoing McKeown minimally invasive esophagectomy (McKeown-MIE) (12), with standardized sarcopenia assessment incorporating muscle mass, handgrip strength, and 4-meter gait speed according to the Asian Working Group for Sarcopenia (AWGS) criteria (5). Our preliminary findings confirmed the adverse impact of sarcopenia on perioperative metabolic stress, postoperative complications, and quality of life recovery (12). Building on this well-characterized prospective cohort, the present study leverages extended long-term follow-up data to further explore the prognostic value of sarcopenia in esophageal cancer.

The primary objectives of this study are: to investigate the impact of sarcopenia on long-term survival in patients with resected esophageal cancer; to integrate sarcopenia into the prognosis prediction system for this disease; and to explore the distinct contribution of muscle mass, muscle strength, and physical performance for survival prediction. This work is anticipated to validate the necessity of standardized sarcopenia assessment and provide prospective evidence to optimize prognosis analysis.

## Methods

### Study design

This prospective cohort study was conducted at the Thoracic Surgery Unit of the Affiliated Cancer Hospital of Zhengzhou University, China. Patient enrollment took place from August 2018 to August 2019, with long-term follow-up continuing until the survival analysis endpoint. The study protocol was approved by the Institutional Ethics Committee of the Affiliated Cancer Hospital of Zhengzhou University (2018127) and was registered in the Chinese Clinical Trial Registry (ChiCTR 1,800,017,792). All enrolled patients provided written informed consent prior to participation. The study was reported in accordance with the strengthening the reporting of observational studies in epidemiology (STROBE) guidelines (13).

### Participants

Patients aged 20 to 80 years with a pathologically confirmed diagnosis of thoracic esophageal cancer who were scheduled to undergo McKeown-MIE, either as primary treatment or following neoadjuvant chemotherapy (NCT), were eligible for inclusion. Exclusion criteria were as follows: advanced tumor stage without indications for surgical resection; severe preoperative comorbidities precluding McKeown-MIE; intraoperative conversion to open thoracotomy; and refusal to provide written informed consent.

### Sarcopenia and pre-sarcopenia assessment

Sarcopenia assessment was performed in a standardized manner on the day before surgery, following the AWGS 2019 consensus (5). The assessment comprehensively covered three core dimensions: appendicular skeletal muscle mass index (ASMI), handgrip strength, and physical performance. All assessment results were blinded to surgeons and outcome evaluators. ASMI was measured using bioelectrical impedance analysis with a multifrequency bioelectrical impedance analyzer equipped with eight tactile electrodes (BCA-IB, Tsinghua Tongfang Co. Ltd., Beijing, China). Low ASMI was defined as < 7.0 kg/m^2^ in males and < 5.7 kg/m^2^ in females. Handgrip strength was evaluated using a digital dynamometer (T. K. K.5401, Takei Scientific Instruments Co. Ltd., Niigata, Japan), with low handgrip strength defined as < 28.0 kg in males and < 18.0 kg in females. Physical performance was assessed using the 4-m walking test, with low gait speed defined as < 0.8 m/s for all patients.

Sarcopenia was diagnosed when low ASMI was present in combination with low handgrip strength and/or low gait speed. Pre-sarcopenia was diagnosed in patients with either (1) low ASMI alone or (2) low handgrip strength and/or low gait speed without low ASMI. Patients diagnosed with sarcopenia or pre-sarcopenia were combined into the sarcopenia risk group. Patients without low ASMI, low handgrip strength, or low gait speed were classified as the normal group.

### Treatment protocol

Clinical tumor staging was determined according to the 8th edition of the Union for International Cancer Control (UICC) TNM classification system. NCT was recommended for patients with nodal disease (cN+) or transmural tumor invasion (cT ≥ 3), consisting of two 3-week cycles of cisplatin plus paclitaxel. Esophagectomy was performed 3–4 weeks after completion of NCT.

All patients underwent thoracolaparoscopic McKeown-MIE with two-field lymph node dissection and cervical hand-sewn anastomosis (12, 14). Perioperative nutritional management was guided by the Nutritional Risk Screening 2002 (NRS 2002): patients with an NRS 2002 score ≥ 5 received mandatory preoperative nutritional support for at least 7–10 days, while those with a score of 3–4 were advised nutritional optimization (15); calorie and protein supply were calculated as 25 kcal/kg/d and 1.5 g/kg/d, respectively (16, 17). Postoperative care included management of fluid and electrolyte balance, nutritional supplementation, pulmonary exercises, and physical rehabilitation.

Postoperative adjuvant chemotherapy or radiotherapy was administered based on the final pathological TNM stage, the patient’s postoperative recovery status, and tolerance to anticancer therapy.

### Endpoints

The primary study outcomes were overall survival (OS) and disease-free survival (DFS). OS was defined as the time from the date of esophagectomy to the date of all-cause death or last follow-up. DFS was defined as the time from esophagectomy to the date of first documented tumor recurrence, distant metastasis, cancer-related death, or last follow-up. The secondary outcome was the incidence of tumor recurrence and/or distant metastasis within 3 years postoperatively, including the site and time of recurrence/metastasis.

All enrolled patients underwent regular postoperative follow-up, conducted via outpatient visits, telephone interviews, and imaging examinations. Follow-up assessments were scheduled at 1, 3, and 6 months postoperatively, and every 6 months thereafter for the first 3 years, with annual follow-up beyond 3 years. Routine follow-up included physical examination, tumor marker detection, chest and abdominal CT, and upper gastrointestinal endoscopy as clinically indicated, to monitor for tumor recurrence and metastasis.

### Statistical analysis

All statistical analyses were performed using IBM SPSS Statistics 22.0 (IBM Corp., Armonk, NY, United States) and R software (version 4.3.1, R Foundation for Statistical Computing, Vienna, Austria). A two-tailed *p*-value < 0.05 was considered statistically significant for all analyses. Categorical variables were presented as frequencies and percentages, and continuous variables as mean ± standard deviation (SD, for normally distributed data) or median and interquartile range (IQR, for non-normally distributed data). Between-group comparisons were conducted using the independent samples t-test, Mann–Whitney U test, Pearson’s χ^2^ test, or Fisher’s exact test, as appropriate.

Propensity score matching (PSM) was performed to balance baseline confounding variables between the sarcopenia risk and normal groups, using a 1:1 matching ratio and a caliper of 0.2. Subgroup analyses were conducted to explore the association between sarcopenia and survival outcomes across different subgroups defined by baseline variables. Kaplan–Meier curves were plotted to estimate OS and DFS, with survival differences compared using the log-rank test. Cox proportional hazards regression models were used to identify independent prognostic factors for survival and to calculate hazard ratios (HRs) and 95% confidence intervals (CIs). In multivariable Cox proportional hazards regression models, the events per variable (EPV)—calculated as the ratio of the number of events to the number of variables—was used to assess model bias and overfitting risk (18). An EPV greater than 10 indicates that the bias and overfitting risk of the models are acceptable (18).

Two nomogram prognostic prediction models were constructed: one incorporating sarcopenia and other independent prognostic factors, and another excluding sarcopenia. The predictive performance of nomograms was evaluated using time-dependent receiver operating characteristic (time-ROC) curves, with the area under the curve (AUC) used to quantify discriminatory ability. Decision curve analysis (DCA) was performed to assess the clinical net benefit of the two nomograms by calculating the net benefit at different threshold probabilities.

To explore the distinct prognostic value of the three sarcopenia criteria—muscle mass, muscle strength, and physical performance—in survival assessment, multivariable Cox proportional hazards regression models and time-ROC curves were first conducted. Random survival forest was then used to evaluate the prognostic performance of the three sarcopenia criteria. SHapley Additive exPlanations (SHAP) was applied to quantify and rank the additive contribution of each criterion to survival prediction by interpreting the random survival forest models, enabling unbiased comparison of their prognostic importance.

## Results

### Participants

**Figure 1:**
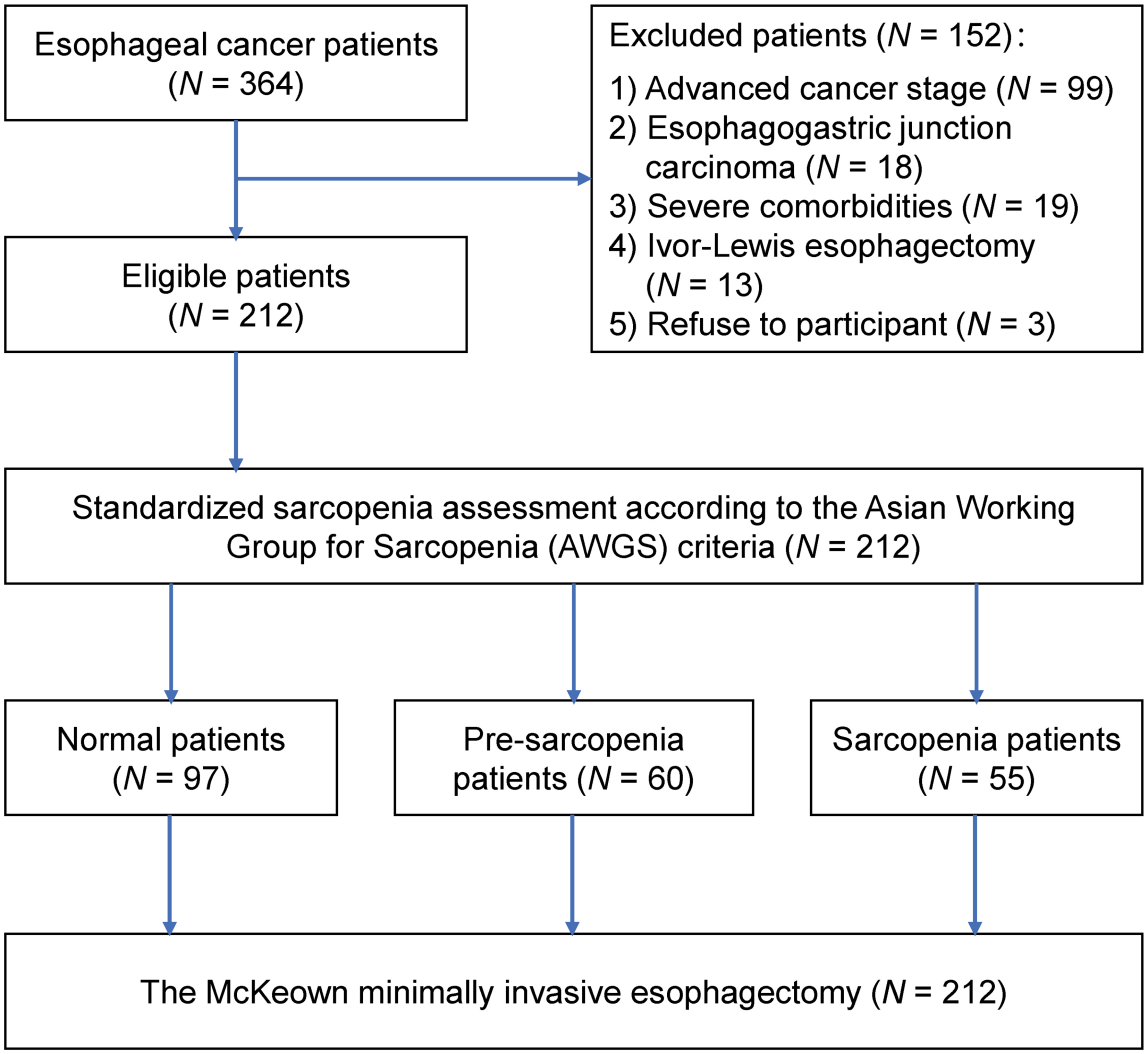
Flowchart for participant recruitment.

During the study period, a total of 364 esophageal cancer patients were prospectively assessed for inclusion. After excluding 152 patients for the reasons noted, the remaining 212 patients were ultimately included (Figure 1). The clinicopathological characteristics of these patients are summarized in Table 1. The mean age was 67.2 years (SD 6.8), with the 95% CI ranging from 52 to 76 years. Of the cohort, 69.6% were male and 30.4% were female. The majority were diagnosed with squamous cell carcinoma (93.0%), and clinical TNM stages were assessed as I (18.3%), II (26.1%), III (54.8%), and IVA (0.9%), respectively. Neoadjuvant chemotherapy was administered to 45 (39.1%) patients, and postoperative adjuvant therapy was administered to 15 (13.0%) patients. The median follow-up for survivors reached 91.8 (89.1–93.4) months.

**Table 1:** Clinicopathological characteristics of the included patients.

Characteristics	Patients with sarcopenia risk				Normal patients (*N* = 97)	*p* values (Sarcopenia risk vs. Normal)
	Total patients (*N* = 115)	Sarcopenia (*N* = 55)	Pre-sarcopenia (*N* = 60)	*p* values		
Demographics						
Age, year	67.2 ± 6.8	68.5 ± 6.4	66.0 ± 7.0	0.11	62.2 ± 6.7	<0.001
Gender						
Male	80 (69.6)	37 (67.3)	43 (71.7)	0.61	65 (67)	0.69
Female	35 (30.4)	18 (32.7)	17 (28.3)		32 (33)	
Smoking history	49 (42.6)	23 (41.8)	26 (43.3)	0.87	45 (46.4)	0.58
Alcohol misuse	28 (24.3)	10 (18.2)	18 (30)	0.14	24 (24.7)	0.95
Comorbidities						
Cardio-cerebral disease	38 (33)	18 (32.7)	20 (33.3)	0.95	19 (19.6)	0.028
Pulmonary disease	17 (14.8)	8 (14.5)	9 (15)	0.95	7 (7.2)	0.083
Diabetes	11 (9.6)	5 (9.1)	6 (10)	0.87	7 (7.2)	0.54
ASA PS grades						
I	67 (58.3)	32 (58.2)	35 (58.3)	0.99	78 (80.4)	0.001
II-III	48 (41.7)	23 (41.8)	25 (41.7)		19 (19.6)	
Pulmonary function						
FEV1 (%predictive value)	97.0 (85.4–109.5)	93.8 (79.4–106.8)	97.7 (91.7–115.8)	0.11	97.5 (83.7–107.5)	0.84
FEV1/FVC (%)	82.8 (78.1–86.8)	80.0 (75.4–90.5)	83.5 (80.7–86.7)	0.17	85.6 (81.4–87.5)	0.057
MVV (%predictive value)	70.0 (57.3–77.0)	64.0 (56.9–73.8)	72.0 (62.2–77.6)	0.058	74.1 (60.7–81.0)	0.057
DLCO-SB (%predictive value)	77.4 (65.4–88.5)	66.5 (58.0–80.3)	81.4 (67.3–89.5)	0.004	86.3 (73.6–97.7)	<0.001
Nutritional parameters						
Body mass index, kg/m^2^	21.4 ± 2.2	20.5 ± 1.9	22.3 ± 2.2	<0.001	24.4 ± 2.6	<0.001
ASMI, kg/m^2^	6.46 (5.67–6.90)	6.36 (5.30–6.63)	6.74 (5.89–7.14)	<0.001	7.19 (5.98–7.75)	<0.001
Handgrip strength, kg	24.8 (20.8–25.8)	22.8 (17.6–24.8)	25.8 (22.9–28.7)	<0.001	27.8 (22.1–29.8)	<0.001
Gait speed, m/s	0.83 (0.79–0.89)	0.80 (0.76–0.84)	0.86 (0.82–0.99)	<0.001	0.95 (0.92–1.02)	<0.001
Malnutrition risk screened by NRS 2002						
No risk	11 (9.6)	3 (5.5)	8 (13.3)	<0.001	38 (39.2)	<0.001
Medium risk	69 (60)	25 (45.5)	44 (73.3)		57 (58.8)	
High risk	35 (30.4)	27 (49.1)	8 (13.3)		2 (2.1)	
Surgical parameters						
Operative time	185 (165–216)	187 (165–227)	184 (165–210)	0.69	180 (160–212)	0.35
Estimated blood loss	100 (70–150)	100 (60–150)	100 (90–150)	0.97	100 (50–175)	0.25
Lymph node dissection, No.	27 (21–33)	27 (20–30)	27 (21–35)	0.62	27 (20–35)	0.74
Radicality						
R0	113 (98.3)	53 (96.4)	60 (100)	0.23	96 (99)	1.00
R1	2 (1.7)	2 (3.6)	0 (0)		1 (1)	
Tumor characteristics						
Tumor location						
Proximal third	9 (7.8)	4 (7.3)	5 (8.3)	0.65	1 (1)	0.066
Middle third	67 (58.3)	30 (54.5)	37 (61.7)		62 (63.9)	
Distal third	39 (33.9)	21 (38.2)	18 (30)		34 (35.1)	
Histology type	0 (0)					
Squamous cell carcinoma	107 (93)	50 (90.9)	57 (95)	0.62	92 (94.8)	0.59
Adenocarcinoma	8 (7)	5 (9.1)	3 (5)		5 (5.2)	
Cancer differentiation						
Well	10 (8.7)	5 (9.1)	5 (8.3)	0.086	10 (10.3)	0.58
Moderately	59 (51.3)	33 (60)	26 (43.3)		49 (50.5)	
Poorly	44 (38.3)	16 (29.1)	28 (46.7)		33 (34)	
Clinical TNM stage						
I	21 (18.3)	8 (14.5)	13 (21.7)	0.010	21 (21.6)	0.99
II	30 (26.1)	9 (16.4)	21 (35)		22 (22.7)	
III	63 (54.8)	37 (67.3)	26 (43.3)		54 (55.7)	
IVA	1 (0.9)	1 (1.8)	0 (0)		2 (2.1)	
Neoadjuvant therapy						
Yes	45 (39.1)	18 (32.7)	27 (45)	0.18	43 (44.3)	0.44
No	70 (60.9)	37 (67.3)	33 (55)		54 (55.7)	
Pathological TNM stage						
0-I	39 (33.9)	14 (25.5)	25 (41.7)	0.14	33 (34)	0.98
II	31 (27)	18 (32.7)	13 (21.7)		27 (27.8)	
III	41 (35.7)	21 (38.2)	20 (33.3)		36 (37.1)	
IVA	3 (2.6)	2 (3.6)	1 (1.7)		2 (2.1)	
Perioperative endpoints						
Overall complications	61 (53)	41 (74.5)	20 (33.3)	<0.001	32 (33)	0.003
Severe complications	17 (14.8)	15 (27.3)	2 (3.3)	<0.001	3 (3.1)	0.004
Postoperative hospital stay, days	10.0 (8–0.0–15.0)	13.0 (8.0–22.0)	9.0 (8.0–11.0)	<0.001	9.0 (7.5–11.0)	0.007
Adjuvant therapy						
Yes	15 (13.0)	6 (10.9)	9 (15.0)	0.52	24 (24.7)	0.029
No	100 (87.0)	49 (89.1)	51 (85.0)		73 (75.3)	

Data are presented as mean ± standard deviation, number (percentage), or median (interquartile range). Group differences were assessed using ANOVA, Pearson’s chi-squared test, Fisher’s exact test, or Mann–Whitney U test. ASA-PS, American Society of Anesthesiologists physical status; ASMI, appendicular skeletal muscle mass index; DLCO-SB, single-breath diffusing capacity of the lung for carbon monoxide; FEV1, forced expiratory volume in 1 s; FVC, forced vital capacity; MVV, maximal voluntary ventilation.

### Sarcopenia assessment and groups

According to the AWGS criteria, preoperative sarcopenia assessment identified 55 patients as sarcopenia, 60 patients as pre-sarcopenia, and the remaining 97 patients as normal. Sarcopenia risk, including sarcopenia and pre-sarcopenia, was identified in 115 patients. The median ASMI, handgrip strength, and gait speed were 6.46 (IQR 5.67–6.90) kg/m^2^, 24.8 (IQR 20.8–25.8) kg, and 0.83 (IQR 0.79–0.89) m/s, respectively. Low ASMI, low handgrip strength, and low gait speed were observed in 74 (34.9%), 74 (34.9%), and 46 (21.7%) patients, respectively. Significant differences in ASMI, handgrip strength, and gait speed were detected in both comparisons of sarcopenia vs. pre-sarcopenia and sarcopenia risk vs. normal patients (Table 1).

### Characteristics of sarcopenia patients

Comparisons of clinicopathological characteristics among the three groups are shown in Table 1. Patients with sarcopenia and pre-sarcopenia were generally comparable in terms of baseline characteristics. However, sarcopenia patients showed poorer lung diffusion capacity (as measured by the single-breath diffusing capacity of the lung for carbon monoxide, DLCO-SB), a higher malnutrition risk according to the NRS 2002 screening tool, and more advanced clinical TNM stage. Compared to normal patients, those with sarcopenia risk showed more advanced age, increased cardio-cerebral diseases, elevated ASA-PS grades, poorer DLCO-SB, and higher malnutrition risk as screened by NRS 2002, but less administration of NCT, with no significant differences in surgical or tumor characteristics. Regarding perioperative outcomes, sarcopenia patients showed increased incidence of overall and severe complications and prolonged hospital stay compared to both pre-sarcopenia and normal patients, while the latter two groups showed comparable perioperative outcomes.

PSM was conducted to balance baseline confounding variables between the sarcopenia risk and non-sarcopenia groups using a 1:1 matching ratio. As shown in Supplementary Table 1, a total of 73 pairs were finally identified after PSM, and patients with sarcopenia risk showed no significant differences in core baseline clinicopathological characteristics.

### 3-year recurrence according to sarcopenia

The 3-year recurrence rates for sarcopenia, pre-sarcopenia, and normal patients were 34.5, 33.3, and 18.6%, respectively (Table 2). Sarcopenia and pre-sarcopenia patients showed no significant differences in overall recurrence, locoregional recurrence, distant metastasis, or other specific recurrences within 3 years after surgery. Compared to normal patients, sarcopenia patients showed an increased incidence of overall recurrence and locoregional recurrence, particularly regional lymph node and anastomotic recurrence, but no significant difference in distant metastases. After PSM, the increased incidence of overall and locoregional recurrences in patients with sarcopenia risk was still observed compared to normal patients.

**Table 2:** Three-year recurrences of the included patients.

Recurrence outcomes	Before propensity score matching						After propensity score matching		
	Patients with sarcopenia risk				Normal patients (*N* = 97)	*p* values	Sarcopenia risk (*N* = 73)	Normal (*N* = 73)	*p* values
	Total patients (*N* = 115)	Sarcopenia (*N* = 55)	Pre-sarcopenia (*N* = 60)	*p* values					
Overall recurrence									
Yes	39 (33.9)	19 (34.5)	20 (33.3)	0.89	18 (18.6)	0.012	26 (35.6)	14 (19.2)	0.026
No	76 (66.1)	36 (65.5)	40 (66.7)		79 (81.4)		47 (64.4)	59 (80.8)	
Locoregional recurrence									
Yes	23 (20.0)	12 (21.8)	11 (18.3)	0.64	8 (8.2)	0.016	13 (17.8)	5 (6.8)	0.044
No	92 (80.0)	43 (78.2)	49 (81.7)		89 (91.8)		60 (82.2)	68 (93.2)	
Distant metastasis									
Yes	24 (20.9)	11 (20.0)	13 (21.7)	0.83	14 (14.4)	0.22	16 (21.9)	10 (13.7)	0.19
No	91 (79.1)	44 (80.0)	47 (78.3)		83 (85.6)		57 (78.1)	63 (86.3)	
Combined recurrence									
Yes	8 (7.0)	4 (7.3)	4 (6.7)	0.90	4 (4.1)	0.37	3 (4.1)	2 (2.7)	0.65
No	107 (93.0)	51 (92.7)	56 (93.3)		93 (95.9)		70 (95.9)	71 (97.3)	
Specific classifications of recurrence									
Regional lymph nodes									
Yes	19 (16.5)	8 (14.5)	11 (18.3)	0.59	8 (8.2)	0.072	12 (16.4)	5 (6.8)	0.071
No	96 (83.5)	47 (85.5)	49 (81.7)		89 (91.8)		61 (83.6)	68 (93.2)	
Anastomosis recurrence									
Yes	13 (11.3)	7 (12.7)	6 (10.0)	0.65	2 (2.1)	0.009	9 (12.3)	2 (2.7)	0.028
No	102 (88.7)	48 (87.3)	54 (90.0)		95 (97.9)		64 (87.7)	71 (97.3)	
Supraclavicular lymph node metastasis									
Yes	6 (5.2)	2 (3.6)	4 (6.7)	0.47	3 (3.1)	0.45	3 (4.1)	3 (4.1)	1.00
No	109 (94.8)	53 (96.4)	56 (93.3)		94 (96.9)		70 (95.9)	70 (95.9)	
Distant organs metastasis									
Yes	20 (17.4)	9 (16.4)	11 (18.3)	0.78	12 (12.4)	0.31	15 (20.5)	9 (12.3)	0.18
No	95 (82.6)	46 (83.6)	49 (81.7)		85 (87.6)		58 (79.5)	64 (87.7)	

Data are presented as number (percentage). Group differences were assessed using Pearson’s chi-squared test or Fisher’s exact test.

### Survival outcomes according to sarcopenia

**Figure 2:**
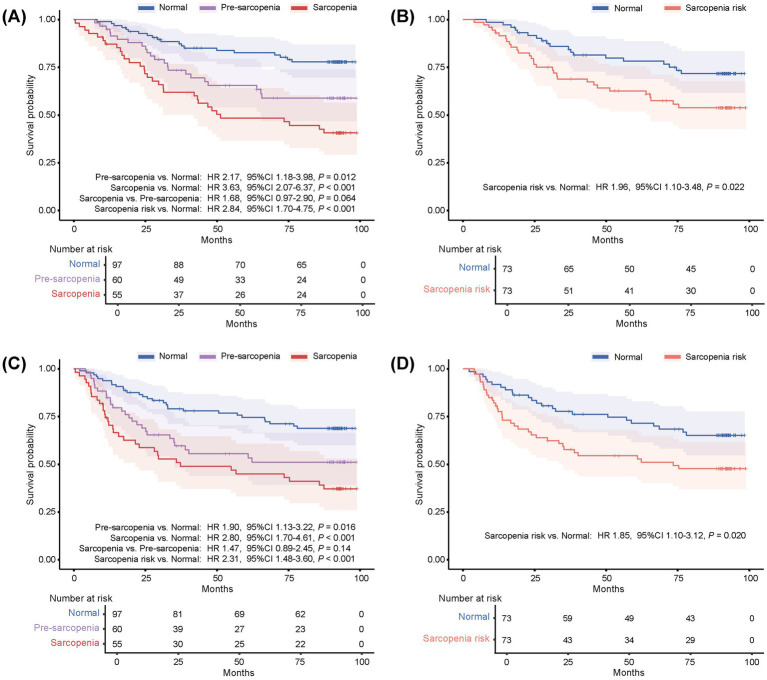
Kaplan–Meier curves illustrating survival profiles. Cox proportional hazards regression models were used to calculate hazard ratios (HRs) and 95% confidence intervals (CIs). **(A,B)** Show overall survival analysis before and after propensity score matching, respectively. **(C,D)** Show disease-free survival analysis before and after propensity score matching, respectively.

As shown in Figure 2, both sarcopenia and pre-sarcopenia patients showed poorer OS and DFS compared to normal patients. No significant difference in OS and DFS was observed between sarcopenia and pre-sarcopenia patients, despite a trend toward poorer survival in sarcopenia patients. When combining sarcopenia and pre-sarcopenia patients, those with sarcopenia risk showed shorter OS and DFS compared to normal patients after surgery. After PSM, the dismal OS and DFS in patients with sarcopenia risk were still observed compared to normal patients.

The results of univariable and multivariable Cox proportional hazards regression models, which included clinical, surgical, and tumor characteristics as well as sarcopenia assessments, are detailed in Table 3. In the multivariable Cox model for OS, increased age, smoking history, cardio-cerebral disease, advanced clinical TNM stage, and sarcopenia were identified as independent predictors of poor OS. In the multivariable Cox model for DFS, smoking history, advanced clinical TNM stage, and sarcopenia were found to be independently predictive of poor DFS. From pre-sarcopenia to sarcopenia, the mortality risk increased, as reflected by the HRs and 95% confidence intervals. Additionally, sarcopenia risk (combining sarcopenia and pre-sarcopenia) was also independently associated with poor OS and DFS. The number of events in the survival analysis was 73 for OS and 89 for DFS. Consequently, the EPV values in the multivariable analysis for OS and DFS were approximately 15 and 30, respectively.

**Table 3:** Analysis of the associated factors for long-term survival.

Characteristics	Comparisons	Overall survival				Disease-free survival			
		Univariable analysis		Multivariable analysis^1^^,^^2^		Univariable analysis		Multivariable analysis^1^^,^^2^	
		HR (95%CI)	*p* values	HR (95%CI)	*p* values	HR (95%CI)	*p* values	HR (95%CI)	*p* values
Demographics									
Age	Per 1 year	1.04 (1.00–1.07)	0.029	1.04 (1.00–1.08)	0.039	1.02 (0.99–1.05)	0.28		
Gender	Male vs. Female	1.56 (0.92–2.66)	0.10			1.37 (0.86–2.19)	0.19		
Smoking history	Yes vs. No	1.68 (1.06–2.67)	0.026	1.75 (1.08–2.84)	0.022	1.65 (1.09–2.51)	0.018	1.61 (1.00–2.58)	0.048
Alcohol misuse	Yes vs. No	1.32 (0.79–2.24)	0.29			1.30 (0.81–2.18)	0.27		
Comorbidities									
Cardio-cerebral disease	Yes vs. No	1.83 (1.14–2.94)	0.012	1.76 (1.09–2.84)	0.021	1.39 (0.89–2.17)	0.15		
Pulmonary disease	Yes vs. No	1.59 (0.73–3.48)	0.25			1.30 (0.60–2.83)	0.50		
Diabetes	Yes vs. No	1.79 (0.86–3.74)	0.12			1.28 (0.62–2.66)	0.50		
ASA PS grades	II-III vs. I	1.73 (1.08–2.74)	0.023			1.41 (0.91–2.17)	0.12		
Pulmonary function									
FEV1 (%predictive value)	Per 1%	1.00 (0.99–1.02)	0.66			1.00 (0.99–1.01)	0.68		
FEV1/FVC (%)	Per 1%	0.97 (0.94–1.01)	0.10			0.97 (0.94–1.00)	0.087		
MVV (%predictive value)	Per 1%	0.99 (0.98–1.01)	0.34			1.00 (0.98–1.01)	0.68		
DLCO-SB (%predictive value)	Per 1%	0.97 (0.95–0.99)	0.001			0.97 (0.96–0.99)	<0.001		
Surgical parameters									
Operative time	Per 10 min	1.05 (1.00–1.10)	0.052			1.04 (1.00–1.08)	0.068		
Estimated blood loss	Per 10 mL	1.01 (1.00–1.02)	0.026			1.01 (1.00–1.02)	0.029		
Lymph node dissection, No.	Per 1 lymph node	1.02 (1.00–1.04)	0.029			1.02 (1.00–1.04)	0.029		
Tumor characteristics									
Tumor location									
Middle third	vs. Proximal third	1.16 (0.36–3.76)	0.80			1.52 (0.47–4.87)	0.48		
Distal third	vs. Proximal third	1.77 (0.54–5.79)	0.35			2.05 (0.63–6.67)	0.23		
Histology type	SCC vs. AD	1.21 (0.44–3.32)	0.71			1.62 (0.81–3.24)	0.17		
Cancer differentiation									
Moderately	vs. Well	1.11 (0.45–2.71)	0.82			1.38 (0.61–3.11)	0.44		
Poorly	vs. Well	1.36 (0.58–3.19)	0.49			1.40 (0.64–3.10)	0.40		
Clinical TNM stage									
II	vs. I	1.20 (0.49–2.96)	0.69	1.52 (0.61–3.77)	0.36	1.56 (0.72–3.39)	0.26	1.43 (0.65–3.24)	0.36
III-IVA	vs. I	2.94 (1.49–5.78)	0.002	2.80 (1.41–5.52)	0.003	2.28 (1.29–4.01)	0.004	2.14 (1.18–3.86)	0.012
Neoadjuvant therapy	Yes vs. No	1.39 (0.87–2.20)	0.17			1.53 (1.01–2.32)	0.047		
Pathological TNM stage									
II	vs. 0-I	1.49 (0.81–2.73)	0.20			1.44 (0.81–2.55)	0.21		
III-IVA	vs. 0-I	1.68 (0.96–2.93)	0.068			2.00 (1.21–3.30)	0.007		
Adjuvant therapy	Yes vs. No	1.52 (0.86–2.70)	0.15			1.97 (1.22–3.18)	0.005		
Sarcopenia assessment^2^									
Sarcopenia classifications									
Pre-sarcopenia	vs. Normal	2.17 (1.18–3.98)	0.012	2.94 (1.58–5.50)	0.001	1.90 (1.13–3.22)	0.016	2.06 (1.21–3.50)	0.007
Sarcopenia	vs. Normal	3.63 (2.07–6.37)	<0.001	3.51 (1.99–6.20)	<0.001	2.80 (1.70–4.61)	<0.001	2.90 (1.75–4.80)	<0.001
Sarcopenia risk	vs. Normal	2.84 (1.70–4.75)	<0.001	3.26 (1.93–5.51)	<0.001	2.31 (1.48–3.60)	<0.001	2.50 (1.60–3.92)	<0.001

1 Multivariable analyses were conducted using Cox proportional hazards regression models with the backward conditional methods. Results are presented as hazard ratios (HRs) with 95% confidence intervals (CIs). Variables with a *p*-value of less than 0.10 in univariate analysis were selected for inclusion in multivariate regression models.

2 Sarcopenia classifications and sarcopenia risk were individually incorporated into the multivariable analyses for model construction and the calculation of HRs. The presented results of multivariable analysis for clinicopathological characteristics were calculated with sarcopenia classifications included as an adjusting factor.

AD, adenocarcinoma; ASA-PS, American Society of Anesthesiologists physical status; DLCO-SB, single-breath diffusing capacity of the lung for carbon monoxide; FEV1, forced expiratory volume in 1 s; FVC, forced vital capacity; MVV, maximal voluntary ventilation; SCC, squamous cell carcinoma.

### Subgroup analysis of survival endpoints

**Figure 3:**
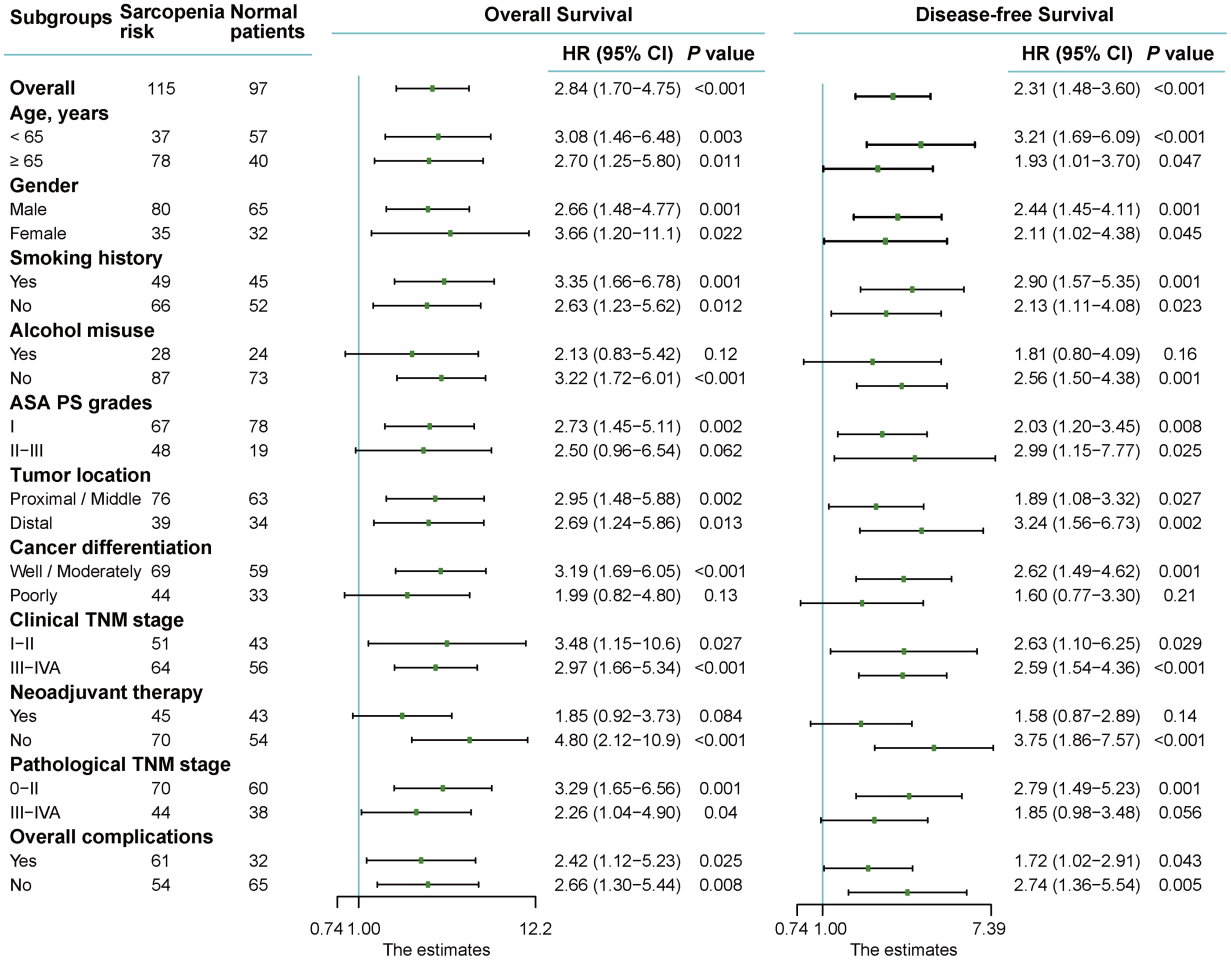
Subgroup analyses examining the association between sarcopenia and survival outcomes across different subgroups defined by baseline variables. ASA-PS, American Society of Anesthesiologists physical status; CI, confidence interval; HR, hazard ratio.

Subgroup analyses according to core baseline clinicopathological variables are presented in Figure 3. Sarcopenia risk consistently showed trends of shorter OS and DFS in subpopulations classified by age, sex, smoking history, ASA PS grades, tumor location, clinical TNM stages, pathological TNM stages, and incidence of complications. Additionally, the association between sarcopenia risk and survival outcomes was attenuated in patients with alcohol misuse, poorly differentiated tumors, neoadjuvant therapy, or advanced pathological TNM stage compared to the matched controls.

### Prediction nomograms for survival outcomes

**Figure 4:**
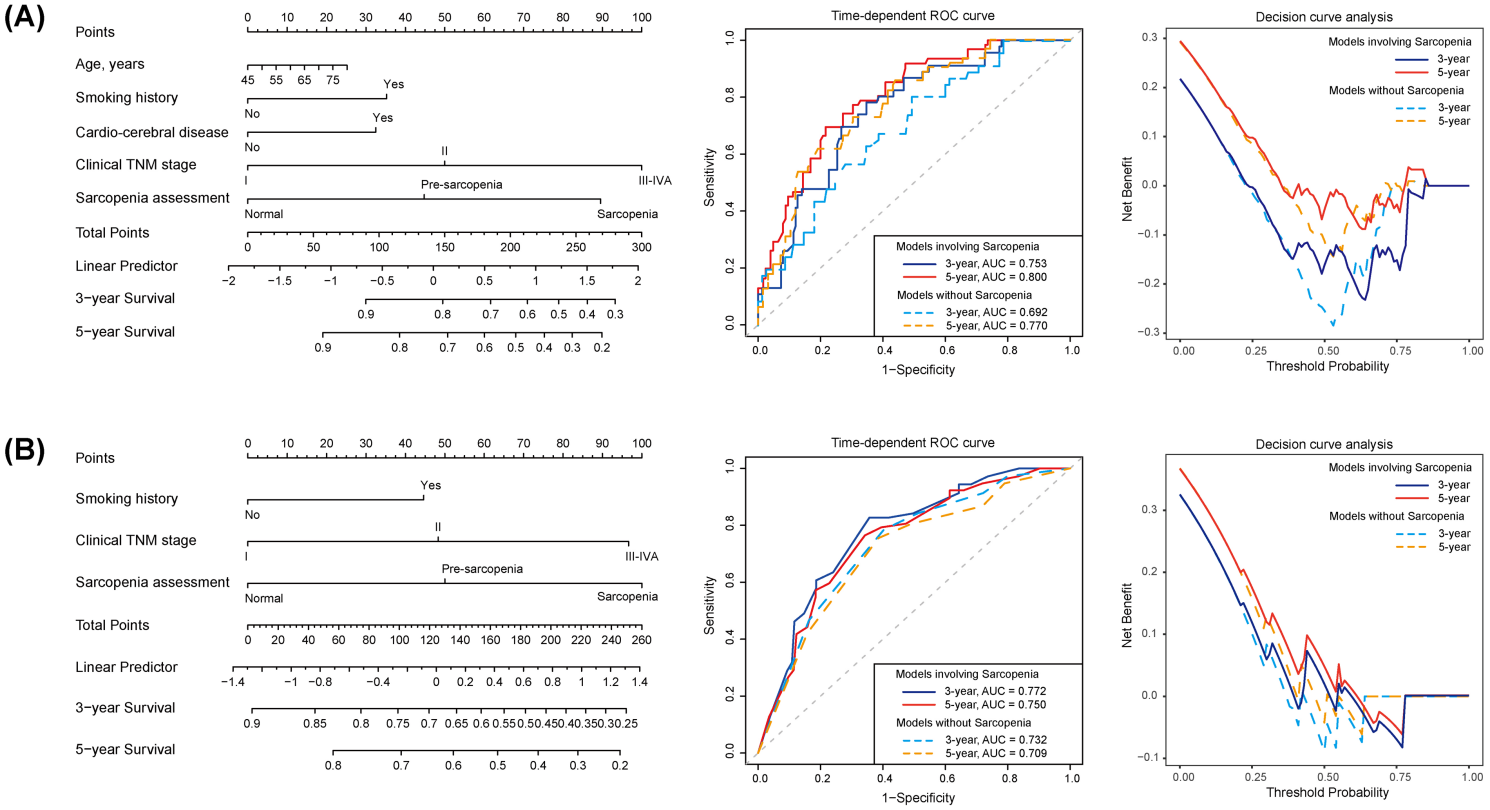
Nomogram models incorporating sarcopenia and other independent prognostic factors for predicting overall survival **(A)** and disease-free survival **(B)**. The predictive performance of the nomograms was evaluated using time-dependent receiver operating characteristic (time-ROC) curves, with the area under the curve (AUC) used to quantify discriminatory ability. Decision curve analysis was performed to assess the clinical net benefit of the nomograms by calculating net benefit at different threshold probabilities. From left to right, the figure presents the nomograms, time-ROC curves, and decision curves.

Nomograms were constructed based on predictive clinicopathological variables and are presented in Figure 4. The sarcopenia classification system contributed significant weight to OS and DFS prediction, comparable to clinical TNM stages. Based on time-ROC curve analysis, compared to nomograms without sarcopenia assessment, the inclusion of the sarcopenia classification system improved predictive performance for both OS and DFS at 3-year and 5-year time points. Based on decision curve analysis, the inclusion of the sarcopenia classification system also improved net benefit at threshold probabilities of 25–60% for OS and 25–70% for DFS at both 3-year and 5-year time points.

### Predictive value of the three dimensions of sarcopenia

**Figure 5:**
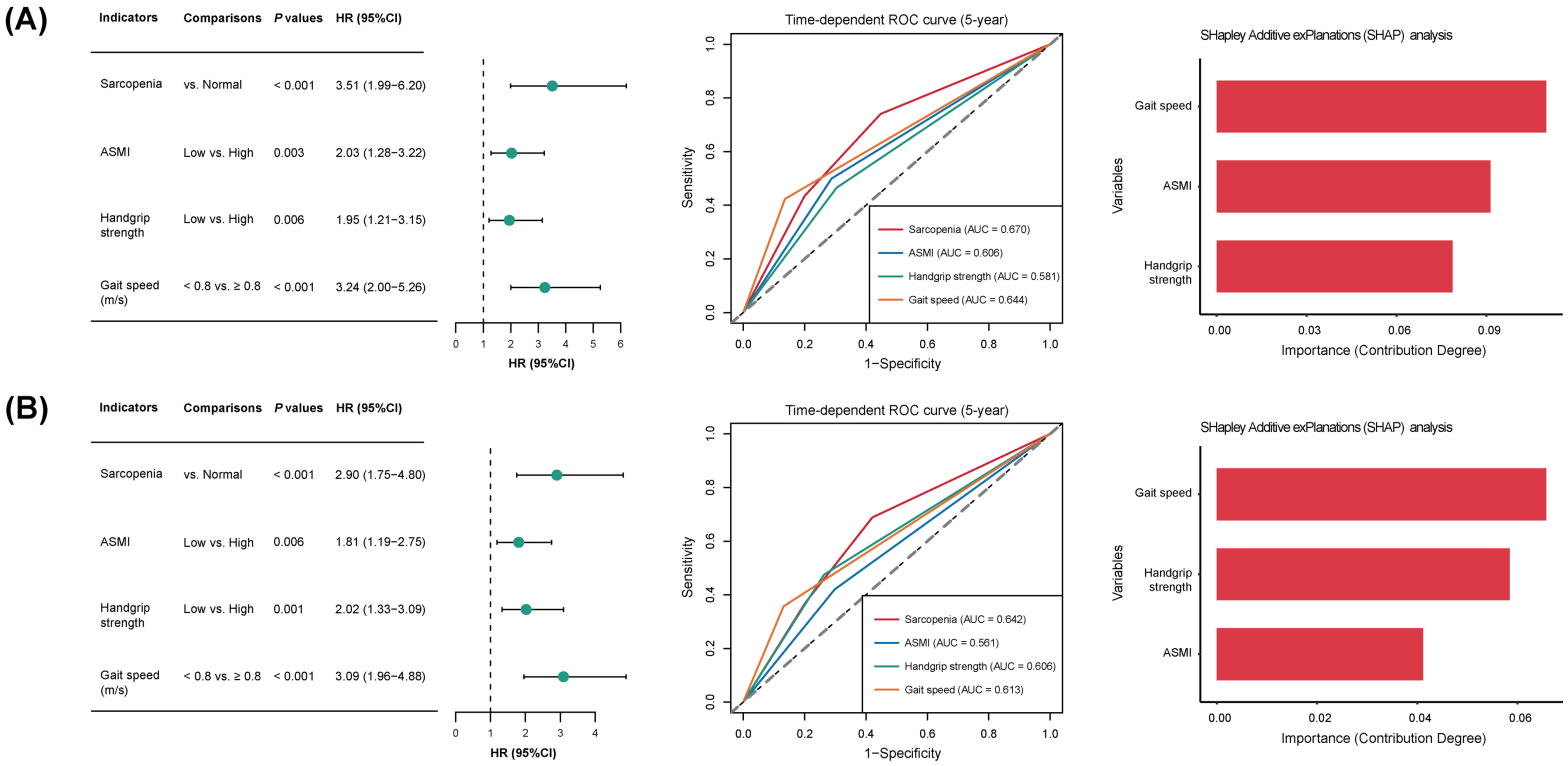
Prognostic value of the three sarcopenia dimensions—muscle mass, muscle strength, and physical performance—for overall survival **(A)** and disease‑free survival **(B)**. Forest plots present the results of multivariable Cox proportional hazards regression models. The predictive performance of muscle indicators was evaluated using time‑dependent receiver operating characteristic (time‑ROC) curves, with the area under the curve (AUC) used to quantify discriminatory ability. SHapley Additive Explanations (SHAP) were applied to quantify and rank the additive contribution of each muscle dimension to survival prediction. From left to right, the figure shows forest plots, time‑ROC curves, and SHAP plots.

After adjusting for core confounding clinicopathological variables as shown in Table 3, the three dimensions of sarcopenia—low ASMI, low handgrip strength, and low gait speed—were independently associated with poor OS and DFS but with differentiated risks (Figure 5). Based on time-ROC curve analysis, their predictive performance for OS was ranked as gait speed > ASMI > handgrip strength, while that for DFS was gait speed > handgrip strength > ASMI. The sarcopenia classification system showed significantly higher predictive performance for OS and DFS than the three individual components. Accordingly, based on SHAP analysis, the additive contribution of each dimension to survival prediction was gait speed > ASMI > handgrip strength for OS and gait speed > handgrip strength > ASMI for DFS.

## Discussion

This study confirmed the independent predictive value of sarcopenia for OS and DFS in esophageal cancer patients undergoing surgical resection. Nomograms incorporating sarcopenia assessment were established to improve prognosis analysis and clinical decision-making. Comprehensive sarcopenia assessment, involving three dimensions, was demonstrated to be necessary for survival prediction.

Sarcopenia have been increasingly recognized as important factor impacting the prognosis of multiple cancer patients, particularly those with respiratory, gastrointestinal, and reproductive malignancies (19–22). Preoperative sarcopenia has been reported to be associated with increased perioperative complications and delayed hospital discharge in gastrointestinal cancers (23, 24). Regarding esophageal cancers, several meta-analyses have validated the adverse impact of sarcopenia on survival profiles in both surgically treated and first-line treated patients (9, 10). Critical limitations of previous studies include retrospective study design, relatively short follow-up, and a sole focus on OS. This prospective cohort study provides long-term follow-up lasting nearly 8 years. Preoperative sarcopenia was demonstrated to impact both OS and DFS for esophageal cancer patients through systematic analysis. Notably, the sarcopenia classification system was investigated for the first time and showed comparable predictive values for survival outcomes to clinical TNM stages. However, we also observed the attenuated association between sarcopenia risk and survival outcomes in patients with poor cancer differentiation, those undergoing NCT, and those with advanced pathological stages. These phenomena indicated that patient prognosis may be increasingly impacted by cancer characteristics as the tumor advances. Therefore, sarcopenia assessment is suggested to be incorporated into prognosis analysis systems established on the basis of cancer stages. We have established nomograms and detected the improvement of sarcopenia assessment for prognosis prediction and clinical-decision making.

Another critical limitation of previous study was the solely assessment of skeletal muscle mass. Most studies retrospective reviewed and analyzed the muscle area at the L3 level to assess muscle mass (9, 10). In recent years, the pectoralis muscle has been increasingly assessed to detect respiratory sarcopenia (25). These limitations stem from the retrospective study design and the lack of routine assessment of muscle strength and physical performance. However, both the Asian and European guidelines have recommended comprehensive assessment of muscle mass, muscle strength, and physical performance for sarcopenia diagnosis, classification, and managements (5, 6). The study by Makiura et al. (26) prospectively assessed sarcopenia according to the AWGS criteria and observed an association between sarcopenia and dismal OS in esophageal cancer patients. However, that study solely reported OS with small sample, without comprehensive subgroup analysis and the establishment of prediction models, and did not compare the three dimensions of sarcopenia assessment (26). Based on our analysis, physical performance, indicated by gait speed, contributed the most to survival prediction, while ASMI and handgrip strength also contributed significantly to predicting OS and DFS, validating the necessity of standardized assessment protocols. On the other hand, both pre-sarcopenia and sarcopenia were demonstrated to adversely impacted OS and DFS. As sarcopenia risk progresses from pre-sarcopenia to sarcopenia, these adverse impacts enlarge. These results indicate that treatment of sarcopenia should be initiated earlier, at the pre-sarcopenia stage, which further advocates for comprehensive assessment of muscle mass, muscle strength, and physical performance.

The mechanisms underlying the association between muscle status and prognosis in cancer patients remain unclear. Sarcopenia can lead to abnormal secretion of myokines related to immune senescence, such as interleukin (IL)-15, IL-17, and IL-6, which modulate the proliferation and function of both innate and adaptive immune cells (27). Previous studies have also reported an association between sarcopenia and impaired proliferation of peripheral mononuclear cells, an increased neutrophil-to-lymphocyte ratio, and disrupted homeostasis of natural killer lymphocytes (28, 29). Esophageal cancer patients with sarcopenia risk may thus have impaired immune killing and surveillance, contributing to increased recurrence and mortality. On the other hand, skeletal muscle is an important peripheral reservoir of proteins, which can be mobilized to supply amino acids to support life systems in treating chronic consumptive diseases, particularly cancers (16, 30). Esophageal cancer patients with sarcopenia risk may have decreased availability of such protein mobilization, which may also contribute to dismal prognosis. More basic research is warranted to clarify these issues. Additionally, despite their differing characteristics and etiologies, sarcopenia has been linked to cachexia and malnutrition (31, 32). These three conditions commonly share the diagnostic criterion of loss of body composition, albeit with distinct parameters (31, 32). Given that esophageal cancer patients often present with poor appetite, eating difficulties, and tumor-related metabolic consumption, loss of body composition—including body weight and muscle mass—is commonly observed in this population (33, 34). Consequently, the connection between sarcopenia, cachexia, and malnutrition may be even stronger in these patients. As a result, the association between sarcopenia and poor survival could be confounded by the effects of cachexia and malnutrition. These concerns raise the question of whether interventions targeting sarcopenia alone would be sufficient to improve the prognosis of esophageal cancer patients, warranting further investigation.

Although sarcopenia is conventionally recognized as an age-related degenerative muscle disease (5), its prevalence among patients with esophageal cancer may extend to all patients due to poor appetite, eating difficulties, and tumor-related metabolic consumption (33, 34). As shown in this study and previous reports (35, 36), esophageal cancer is also characterized by older age. Furthermore, the updated AWGS 2025 Consensus has expanded the diagnosis of sarcopenia to include middle-aged adults (50–64 years) (37). Therefore, standardized sarcopenia assessment is recommended for definitive diagnosis and classification during the initial evaluation of all patients with esophageal cancer. Pre-sarcopenia should prompt effective interventions to prevent further deterioration and improve muscle profiles. Protein supplementation combined with resistance exercise has been demonstrated to improve muscle volume and function and could be effective methods for muscle promotion (38, 39). For esophageal cancer patients with sarcopenia risk, muscle interventions should be administrated throughout treatments. Neoadjuvant therapies, especially chemoradiotherapy, have been demonstrated to cause significant muscle loss and should be considered as an important period for muscle interventions (40). Esophagectomy, including the reconstruction of the upper gastrointestinal system, have been demonstrated to cause continuous muscle loss until 12 weeks after surgery, which can also be an important period for preventing the incidence and deterioration of sarcopenia (41). Additionally, the long-term muscle promotion after surgery have the potential to improve survival outcomes considering its roles in immune modulation and nutrition preservation, which requires clinical validation.

Several limitations should be considered when interpreting our findings. Despite the prospective study design and long-term follow-up, the sample was relatively small and was recruited from a single institution. We only analyzed Asian esophageal cancer patients undergoing McKeown-MIE with squamous cell carcinoma as the predominant pathological type. The prognosis prediction nomograms integrating sarcopenia classifications have not been externally validated. Therefore, the main findings of this study warrant validation in different regions and for different populations. Additionally, changes in sarcopenia status during neoadjuvant therapy and follow-up after surgery were not reported due to the lack of dynamic assessments. Since this study is observational, the benefit of interventions for sarcopenic and pre-sarcopenic patients should be further investigated.

## Conclusion

Both pre-sarcopenia and sarcopenia were demonstrated to be independent predictors of recurrence and survival in esophageal cancer patients undergoing McKeown-MIE. Standardized sarcopenia assessment, including muscle mass, muscle strength, and physical performance, is recommended to achieve definitive diagnosis and classification of sarcopenia risk. The established prognosis prediction nomograms integrating sarcopenia classifications are anticipated to improve clinical decision-making and warrant external validation. Sarcopenia emerges as a promising target for improving the prognosis of esophageal cancer patients and should be further investigated.

## Data Availability

The original contributions presented in the study are included in the article/Supplementary material, further inquiries can be directed to the corresponding authors.
