# Supplementary material for: Standardized sarcopenia assessment should be incorporated into prognosis analysis of esophageal cancers: a prospective cohort study lasting 8 years
Source: Front Nutr. 2026 Jun 12;13:1865456. doi: 10.3389/fnut.2026.1865456 (PMC13303378; doi:10.3389/fnut.2026.1865456)
Supplement: Supplementary file 1 [file Table_1.DOCX]

**Supplementary Table 1. Baseline clinicopathological characteristics of the included patients after propensity score matching.**

| Characteristics | Sarcopenia risk  (*N* = 73) | Normal patients  (*N* = 73) | *P* values |
| --- | --- | --- | --- |
| ***Demographics*** |  |  |  |
| Age, year | 64.9 ± 7.0 | 63.8 ± 6.4 | 0.32 |
| Gender |  |  |  |
| Male | 56 (76.7) | 49 (67.1) | 0.20 |
| Female | 17 (23.3) | 24 (32.9) |  |
| Smoking history | 37 (50.7) | 35 (47.9) | 0.74 |
| Alcohol misuse | 20 (27.4) | 19 (26.0) | 0.85 |
| ***Comorbidities*** |  |  |  |
| Cardio-cerebral disease | 13 (17.8) | 19 (26.0) | 0.23 |
| Pulmonary disease | 3 (4.1) | 4 (5.5) | 1.00 |
| Diabetes | 8 (11.0) | 6 (8.2) | 0.57 |
| ASA PS grades |  |  |  |
| I | 57 (78.1) | 54 (74.0) | 0.56 |
| II-III | 16 (21.9) | 19 (26.0) |  |
| ***Pulmonary function*** |  |  |  |
| FEV1 (%predictive value) | 97.2 (91.1-112.8) | 97.1 (84.7-108.8) | 0.45 |
| FEV1/FVC (%) | 83.1 (79.4-86.6) | 85.6 (79.4-88.6) | 0.36 |
| MVV (%predictive value) | 72.0 (65.0-78.0) | 71.7 (61.2-80.8) | 0.99 |
| DLCO-SB (%predictive value) | 80.0 (65.9-89.2) | 84.5 (70.0-95.1) | 0.16 |
| ***Surgical parameters*** |  |  |  |
| Operative time | 185 (165-212) | 182 (157-207) | 0.21 |
| Estimated blood loss | 100 (65-150) | 100 (50-150) | 0.26 |
| Lymph node dissection, No. | 25 (20-30) | 27 (20-32) | 0.24 |
| Radicality |  |  |  |
| R0 | 71 (97.3) | 72 (98.6) | 1.00 |
| R1 | 2 (2.7) | 1 (1.4) |  |
| ***Tumor characteristics*** |  |  |  |
| Tumor location |  |  |  |
| Proximal third | 3 (4.1) | 1 (1.4) | 0.33 |
| Middle third | 47 (64.4) | 42 (57.5) |  |
| Distal third | 23 (31.5) | 30 (41.1) |  |
| Histology type |  |  |  |
| Squamous cell carcinoma | 67 (91.8) | 69 (94.5) | 0.51 |
| Adenocarcinoma | 6 (8.2) | 7 (9.6) |  |
| Cancer differentiation |  |  |  |
| Well | 6 (8.2) | 7 (9.6) | 0.92 |
| Moderately | 37 (50.7) | 37 (50.7) |  |
| Poorly | 29 (39.7) | 27 (37) |  |
| Clinical TNM stage |  |  |  |
| I | 15 (20.5) | 18 (24.7) | 0.74 |
| II | 17 (23.3) | 15 (20.5) |  |
| III | 40 (54.8) | 39 (53.4) |  |
| IVA | 1 (1.4) | 1 (1.4) |  |
| Neoadjuvant therapy |  |  |  |
| Yes | 35 (47.9) | 28 (38.4) | 0.24 |
| No | 38 (52.1) | 45 (61.6) |  |
| Pathological TNM stage |  |  |  |
| 0-I | 27 (37.0) | 22 (30.1) | 0.58 |
| II | 17 (23.3) | 21 (28.8) |  |
| III | 27 (37.0) | 28 (38.4) |  |
| IVA | 2 (2.7) | 2 (2.7) |  |
| ***Adjuvant therapy*** |  |  |  |
| Yes | 13 (17.8) | 14 (19.2) | 0.83 |
| No | 60 (82.2) | 59 (80.8) |  |

Data are presented as mean ± standard deviation, number (percentage), or median (interquartile range).

Group differences were assessed using ANOVA, Pearson’s chi-squared test, Fisher’s exact test, or Mann–Whitney U test.

Abbreviations: ASA‑PS, American Society of Anesthesiologists physical status; DLCO-SB, single-breath diffusing capacity of the lung for carbon monoxide; FEV1, forced expiratory volume in 1 second; FVC, forced vital capacity; MVV, maximal voluntary ventilation.
